# P-1483. Clinical Features and Outcomes of KPC and NDM Carbapenemase-Producing Enterobacterales Infection in the Era of Avibactam

**DOI:** 10.1093/ofid/ofae631.1653

**Published:** 2025-01-29

**Authors:** Fabián Herrera, María V Leone, Diego Torres, Rocío Rojas, Florencia Bues, Andrés N Rearte, Elena Temporiti, Marcia Querci, Jimena Carlos, Federico Nicola, Silvia Relloso, Jorgelina Smayevsky, Pablo Bonvehi

**Affiliations:** Centro de Educación Médica e Investigaciones Clínicas, CEMIC, BUENOS AIRES, Ciudad Autonoma de Buenos Aires, Argentina; Hospital Universitario CEMIC, Buenos Aires, Ciudad Autonoma de Buenos Aires, Argentina; Centro de Educación Médica e Investigaciones Clínicas, CEMIC, BUENOS AIRES, Ciudad Autonoma de Buenos Aires, Argentina; Hospital Universitario CEMIC, Buenos Aires, Ciudad Autonoma de Buenos Aires, Argentina; Hospital Universitario CEMIC, Buenos Aires, Ciudad Autonoma de Buenos Aires, Argentina; Vicente Lopez Municipality, Buenos Aires, Ciudad Autonoma de Buenos Aires, Argentina; Hospital Universitario CEMIC, Buenos Aires, Ciudad Autonoma de Buenos Aires, Argentina; Hospital Universitario CEMIC, Buenos Aires, Ciudad Autonoma de Buenos Aires, Argentina; Hospital Universitario CEMIC, Buenos Aires, Ciudad Autonoma de Buenos Aires, Argentina; Hospital Universitario CEMIC, Buenos Aires, Ciudad Autonoma de Buenos Aires, Argentina; Hospital Universitario CEMIC, Buenos Aires, Ciudad Autonoma de Buenos Aires, Argentina; Hospital Universitario CEMIC, Buenos Aires, Ciudad Autonoma de Buenos Aires, Argentina; Hospital Universitario CEMIC, Buenos Aires, Ciudad Autonoma de Buenos Aires, Argentina

## Abstract

**Background:**

Carbapenemase-producing Enterobacterales infections (CPEi) often have poor prognosis and high mortality. However, no local studies compare KPC and NDM-CPEi treated with ceftazidime-avibactam (CA) or ceftazidime-avibactam plus aztreonam (CA+ATM).

**Methods:**

Prospective cohort study. We included episodes of KPC and NDM-CPEi in patients admitted to a university hospital (from August 2018 to April 2023) who received definitive antibiotic treatment (AT) with CA or CA+ATM, respectively. We compared clinical characteristics and outcomes with 30-day follow-up. We performed a logistic regression for 30-day mortality analyses.

**Results:**

One hundred and fifty-six episodes of CPEi were included (117 KPC and 39 NDM). Median APACHE II score was 15 (10-21) vs. 16 (10-20.5), p=0.755. Patients were immunocompromised in 53.8% vs. 41%, p=0.165. Bacteremia was present in 47% vs. 59%, p=0.196, and urinary and abdominal were the most frequent clinical sources of infection (44.4% vs. 38.5%, p=0.513; and 9.4% vs 15.4%,p=0.224; respectively). Klebsiella spp. was the main isolate in both groups (75.2% vs. 87.2%, p=0.117). Empirical AT was appropriate in 59.8% vs. 56.4%, p=0.707; and they received empirical CA in 45.3% vs 38.5%, p=0.456. Septic shock occurred in 24.8% vs. 35.9%, p=0.179. Seven and 30-day mortality and infection-related mortality were 3.4% vs. 0%, p=0.312; 13.7% vs. 25.6%,p=0.082; and 4.3% vs. 12.8%, p=0.071. APACHE II >20 [OR 5.9 (95% CI 1.97-17.98), p=0.002], and skin and soft tissue infection [OR 6.02 (95% CI 1.22-29.62), p=0.027] were independently associated with 30-day mortality, while the 7-day clinical response was a protective factor [OR 0.04 (95% CI 0.004-0.405) p=0.006].

**Conclusion:**

In patients with KPC and NDM-CPEi treated with CA or CA+ATM, clinical features and outcomes were similar. Mortality was associated with the severity and the source of infection while the seven-day clinical response was a protective factor. Our data support the role of CA AND CA+ ATM in these types of infections.

**Disclosures:**

**Diego Torres, Medical Doctor**, Gador: Honoraria|GSK: Advisor/Consultant|Khight Therapeutics: Honoraria|MSD: Honoraria

